# On the Management of Hanwell Lunatic Asylum

**Published:** 1849-07-01

**Authors:** 


ART. V.—*The Fourth Report of the Committee of Visitors of the County Lunatic Asylum, at Hanwell.* January Quarter Sessions, 1849. London: Norris. 1849.

THE Hanwell Lunatic Asylum was opened for the reception of patients on the 16th May, 1831, and enjoys the pre-eminence of having carried the "non-restraint system," as it is termed, to the greatest possible extent; in fact, neither strait-waistcoat, muff, body-staps, leg-locks, coercion-chair, or any kind of instruments of restraint are now admitted within its walls.

On the 21st September, 1839, every form of instrumental restraint was discontinued; and from that period to the present, every annual report has recorded in terms of the highest commendation the success which has attended the new plan. From the opening of the Asylum in 1831, to December, 1848, there have been admitted 3142 patients, of whom 768 appear to have been cured, 160 relieved, and 1251 have died. On the 31st of December, 1848, there were in the Asylum, 967 patients, and 94 officers and attendants, making a total of 1061 inmates. The entire establishment is under the immediate control and supervision of a committee of twenty magistrates and country gentlemen, who, to their honour be it spoken, attend the Asylum daily, to discharge the onerous and responsible duties of their office.

Looking at the high position which the Hanwell Lunatic Asylum aspires to, amidst other contemporary asylums, we are tempted to make some observations upon its constitution and government, and the mode in which it is conducted. We are in the first place surprised to observe, that the committee of management assumes to itself a jurisdiction which should be purely professional. From the Report before us, we find that the committee not only controls the financial and domestic arrangements of the establishment, but adjudicates upon the admission and the discharge of patients, and thus constitutes itself, *de facto*, a medical board. We have no doubt but that the members of the committee exercise their judgment in the most conscientious manner, and that they allow themselves to be influenced by the opinion of the medical officers; but the principle of non-professional interposition in the management of any public hospital or asylum is in itself objectionable. We cannot understand country gentlemen examining, quarterly, every patient in the establishment, and determining which cases shall be admitted, and which discharged. It appears to us an anomaly. These are strictly professional duties, which should be delegated exclusively to the medical officers of the institution. The Report before us states that—

"A quarterly examination of every patient in the Asylum by the COMMITTEE, with records of their proceedings and remarks, is no ordinary obligation, but has been cheerfully and regularly fulfilled. And their own spontaneous regulations, *that every patient admitted shall be introduced to the* COMMITTEE, *and that none shall be discharged without similar intercourse,* all multiply the attentions demanded, whilst obviously those regulations for the protection of the patients must be applauded by the judicious and humane.

"Well to regulate such an establishment also, peculiar experience and discernment are to be desired. Numerous as are the patients, and classified as they may be, on their arrival and during their stay, under the usual heads adopted by medical men, *there exists generally a peculiarity in every individual case—and special attentions, where the peculiarity is ascertained, will allay greatly the wretchedness of the insane,* and assuredly soothe and tranquillize the patient, and often accelerate the cure. *By frequent and gentle intercourse on the part of the members of the* COMMITTEE, confidence and respect are produced, and they are perpetually gratified by a recognition of themselves on the part of the patients, as their benefactors and their friends."

We concur with the humanity and spirit of these remarks, but we believe the "peculiarity which exists in every individual case" can be only fairly appreciated by a professional eye, and we doubt whether "the frequent and gentle intercourse" of unprofessional visitors can be of any advantage to the insane. We cheerfully admit that a frequent and systematic general supervision is not only desirable, but necessary, in so large an establishment as Hanwell; but here the labours of the committee should legitimately end.

In the constitution of the Hanwell Asylum, we are also struck with the paucity of medical officers attached to it. There appear in round numbers to be about 500 patients on the male, and 500 on the female, side; yet there is only one resident medical officer attached to each department, and one visiting physician for the whole establishment. The inefficiency of so small a medical staff is obvious. If we look across the Channel, we find in Paris, that the Salpétrière, with its thousand patients, has four times the number of visiting physicians, and ten times the number of resident medical officers. The disproportion between the sane and the insane is here so great, that it is impossible under such a system to bring any moral influence to bear upon the afflicted multitude. Then, again, this Asylum costs the county 26,500*l.* per annum, (as appears by the present report,) and yet does nothing of any importance for the benefit of this department of science. We remember with pleasure the lectures of Dr. Conolly, and we hail with peculiar satisfaction his appointment, by the President of the College of Physicians, to deliver the Croonean Lectures.

Such an asylum as Hanwell ought to do something more for the benefit of science. There ought to be a more numerous medical staff, and a permanent *clinique* attached to such an institution. We have statistical tables before us, giving the number of patients admitted, and discharged—cured, relieved, and dead; but where are its pathological reports? Since the opening of the Asylum, 1251 patients have died, yet the post-mortem examinations, when such have taken place, have been made privately, and neither student nor professor been benefited by witnessing the result. The County Asylum of Hanwell, supported largely as it is by county rates and parish assessments, is as much a hospital as St George's, or St. Bartholomew's, and ought to have a medical staff as numerous and efficient as these, or any other metropolitan hospitals. While charity might thus be administered upon the highest principles of Christian benevolence, something ought to be done to advance our knowledge of science, and thereby enable us to relieve the afflictions of suffering humanity.

The Report before us, as usual, expresses the highest satisfaction at the enlightened, humane, and effective system of non-restraint which has, the committee states, rendered the Hanwell establishment a model to be imitated by all other public asylums. We must not, however, deceive ourselves. In all such institutions, there will occasionally be found dangerous, ferocious, and rampant lunatics, whom it is necessary, while the furor lasts, to restrain. The paroxysm may not last long, but while it is at its height, the unfortunate patient, for his own sake, as well as for the safety of other persons, must in some way be secured. This is done at Hanwell, not by the use of the hand-strap and body-belt, or the strait-waistcoat, or any other mechanical means; but instead of these, human restraint is substituted, and solitary confinement in the padded room. The Report of the Commissioners in Lunacy is perfectly clear upon this point. Those who profess the entire disuse of restraint, employ manual force and seclusion as parts of their methods of management; but, observe the Commissioners—"In those cases where the patient is overpowered by a number of keepers holding his arms and hands during a paroxysm of violence, restraint is manifest. Even in those cases where the patient is forced into a cell by manual strength, and prevented from leaving it until his fit of excitement shall have passed, it is difficult to understand how this can be reconciled with the profession of abstaining from all restraint whatever, so as to be correctly termed 'non-restraint.' "—*Metropolitan Report,* 1848. Upon these grounds, Foville and other eminent authorities, have designated the "non-restraint system" at Hanwell "a fiction," because, after all, it amounts only to the substitution of one mode of restraint for another mode of restraint. This may be true, but the most humane system should be adopted, and from the time when Pinel emancipated the unhappy lunatic from the chains, bolts, and bars which thrust "the iron into his soul," every attempt has been made to abolish restraint altogether, and certainly in no establishment has this effort been carried out more successfully than at Hanwell. If it be "a fiction," it is better that such a fiction should exist, than the cruel reality of modes of restraint which inflicted torture under the old system, and which precluded, in most cases, any chance of recovery. When the Commissioners visited the asylum on the 12th July, 1848, there were 967 patients in the establishment, of whom three were in seclusion, and fifty-nine under medical treatment.

In Hanwell Asylum, it should be added, there is less necessity for restraint or seclusion than in many other institutions of the kind, because the majority of cases there are chronic and incurable, and such patients, speaking generally, are not very liable to paroxysms of excitement.

From the Report before us, we find that during the year 1848, there were 165 patients admitted, of whom 29 were discharged cured, 7 relieved, and 77 died. This appears to be a large proportion of deaths; but it is to be remembered that many of the cases were incurable upon admission. Hence, also, the proportion of cures is much smaller than might be expected under the present improved system of treatment. The per centage of cures from the opening of the asylum in 1831 to the end of the year 1848 is estimated at only 6·36, and the per centage of deaths at 10·02. The committee again calls upon the local authorities to send recent, and not old and chronic cases to the asylum.

"Edified by the confirmed experiences of another year, your committee would renew to the parochial authorities of the county the entreaties that have formerly been made, that *recent* and *not chronic* patients should be sent to the asylum. The concurrent testimony of all medical men experienced in the treatment of the insane pronounces that, generally, early cases are curable; and the facts of the past year demonstrate that rarely has a case occurred in which an early cure has not followed an early treatment; while prudence and kindness should unite to prevent the selecting too frequently, old, hopeless, and diseased subjects, to occupy the wards of the asylum, and to absorb an attention from the attendants and the officers, which might be so much better and availingly applied."

We have frequently reflected with wonder at the fact, that notwithstanding the numerous munificent institutions which exist in this country, where the hand of Christian charity dispenses her favours so liberally, there is not any hospital in the metropolis for the reception of incurables. We have often been called upon to attend poor miserable beings, who, on account of their disease being incurable, have been refused admission into any hospital, and sent home to die in a wretched attic, where the want of the most ordinary necessities has aggravated and embittered their dying sufferings. It would be well to consider whether it might not be advisable, when the two county asylums are open, to make Colney Hatch an asylum for curative treatment, and Hanwell an asylum for incurables, or *vice versâ.*

The most interesting portion of the present Report is the account which the committee gives of the educational improvement of the patients in Hanwell Asylum:—

"The last Annual Report referred to the great number of human beings who were demented, imbecile, and idiotic; also to the efforts made for some education of the insane by Mr. Gaskell, the active and intelligent medical officer of the Lancaster County Asylum, now most worthily promoted to the honourable situation of a Commissioner of Lunacy; and also in the Surrey County Asylum, and to the surprising benefits that had crowned the attempts ingeniously and generously made in France, Prussia, Switzerland, and America, under most illustrious auspices, and the direction of M. Séguin, Dr. Voisin, and M. Valée, of Dr. Guggenbühl, and others, justly distinguished for a charity and skill beyond all praise. It also announced a design to make an experiment, as to which no sanguine expectations could be reasonably cherished, but which it appeared to be a duty to attempt. For the plan and their resolutions, the committee refer to their last Annual Report. There it was announced that no patient would be compelled to attendance—that attendance should not interfere with the recreations or customary useful labour of those patients who were productively or healthfully employed—and that the schools would not be designed merely to teach patients to read, write, and similar matters, but for the awakening and improving the intellectual state of the imbecile and idiotic, and for the alleviation and gratification, by instruction in natural history, geography, and general knowledge, of those patients who were already partially educated and instructed, and so as to excite, relieve, and recreate, as well as to inform their minds. To the superintendence of that attempt, your committee have devoted themselves during the year, and consider that their limited hopes have not been disappointed, and that they have good reason to be content. With pleasure they acknowledge the cordial co-operation of the medical authorities, the chaplain, the matron, and the other officers, all of whom have shown much generous interest.

"Fortunate, too, have the committee been in the appointment of Mr. Frederick Waite, late of Exeter, as the master of the male school; and of his daughter, Miss Charlotte Waite, as the competent and eligible mistress of the female school. Ordinary expressions, as applied to the governors and governesses of schools, would convey no adequate ideas of the capability of both of them for the situations which they occupy, and of the intelligence, zeal, good temper, and perseverance which both have manifested, and which have won for them just and universal confidence and esteem. The schools were opened with the commencement of the year. The patients have been selected by the medical officers of each department. Daily in the forenoon and afternoon the pupils are assembled. To the much approved methods adopted in France and Switzerland, recourse has been had. Besides the usual occupation in the forenoon and afternoon, an evening male writing-class is periodically assembled, and thither the attendants who wish for improvement also repair. Weekly, from 60 to 70 male and female patients congregate in the chapel, and, with suitable tuition, form a musical class, which is very worthily popular, and by which the religious services have been rendered far more harmonious and pleasant, and would not discredit the congregations assembled in many a parochial edifice or cathedral choir. Not limited to those performances, Mr. Waite has excited much interest, and gives much satisfaction by evening lectures delivered on physical geography, entomology, and the cultivation and properties of plants, well illustrated, and which sometimes 150 patients and persons have been gratified to hear; and they form subjects for hope, for remembrance, and for conversation, and present a pleasing and propitious novelty in the management of the insane. Your committee hope, that on this interesting subject, the mention of some statements made at the desire of their chairman, by Mr. Waite and his daughter, will prove acceptable to the court; and especially as the annual reports obtain an extended circulation and reference, and as detailed information on such topics must prove exemplary and useful. Such statement of Mr. Waite contains the following information :—

"'I forward you a condensed report of the patients attending the male school during the past year. Since we commenced in January, 1848, 64 patients have attended the school; with respect to their ages—

| | | | | |
|---|---|---|---|---|
| "' 5 | are above | 10 | and under | 20 |
| 19 | " | 20 | " | 30 |
| 15 | " | 30 | " | 40 |
| 8 | " | 40 | " | 50 |
| 7 | " | 50 | " | 60 |
| 2 | " | 60 | " | 70 |

"'Of these patients, 31 are epileptic, attended with mania. 9 of the number under various stages of imbecility. 20 are insane, subject to periods of excitement. 3 are congenital idiots; and there were 2 recent cases of mania, who have been cured and left the asylum.

| | | |
|---|---|---|
| " '16 | of the patients | can read and write fairly. |
| 18 | " | can read and write imperfectly. |
| 9 | " | can read a little, and not write. |
| 6 | " | never speak, but anxiously watch the lessons, particularly those given to others on the slate; they can also write words on a slate. |
| 4 | " | are of a still lower grade, and amuse themselves with pictures, and slate and pencil. |
| 3 | " | hitherto taught only a few gymnastic exercises. |

"The few following prominent cases may crave your consideration:—

" 'C. C., 27 years of age, Epileptic, attended with frequent Mania. This patient has attended the school from its commencement; he is usually attentive and obedient, and very desirous of obtaining instruction and information. During his worst periods of excitement, I have rarely failed to tranquillize him during school time.

" 'J. C., 31, Mania attended with Imbecility. He is always mild and gentle; has attended the school eight months; he is very fond of reading and writing, in both of which he has greatly improved; he has again resumed his attendance, after a long and severe illness.

" 'W. B., 50, Occasional Violent Mania. He has attended the school with tolerable regularity the last six months; reads and writes, and is very fond of arithmetic; even under considerable excitement, he becomes tranquil on giving him a sum to work.

" 'E. D., 33. (Frenchman.) He has attended the school from its commencement. Epileptic, with recurrent periods of excitement, and paralyzed; the improvement of this man is very considerable; his reading was very imperfect, and his writing worse; in both he has wonderfully improved, spells exceedingly well, and has acquired much information.

" 'D. D., 28, Insane. Attended the school for three months with considerable effect; he was afterwards employed out of doors, till he was discharged cured. During the latter period, he attended the evening classes.

" 'P. H. Q., 24. A very remarkable case of Melancholia. This patient never speaks, but will copy for me letters of a commercial nature, bills of lading, &c.

" 'J. H., 34, Epileptic, attended with Imbecility. This patient has been in the school for some months without speaking. I saw him on one occasion apparently reading a piece of newspaper with much attention; and I inquired of him, if he would read a little book to me if I gave him one? He replied, Yes. Since that period, he constantly reads and writes during school hours.

"'C. W., 19, Epileptic, with Imbecility. He has attended the school from its commencement, and has very greatly improved in reading and writing, &c.; indeed, I may add, what he does know, he has wholly acquired in the school.

"'H. M., 30, Imbecile. He has attended the school from its commencement, and notwithstanding his imbecility, has been taught to write and read; owing to his having a very imperfect knowledge of sounds, he has not acquired the latter with the same facility as writing; he is exceedingly fond of pictures, and by their aid in teaching him to read, the labour has been greatly lessened.

"'J. O. D., 25, Epileptic, with occasional Mania. An exceedingly attentive, well-behaved young man; reads extremely well, writes tolerably; numbers perplex him; the latter I find to be almost invariably the case, with the epileptic patients especially.

"'J. L., 28, Idiot. He is now learning to write, and with great hope of success; he has hitherto been regarded as incapable of being taught.

"'B. F., 30, Imbecility in a low degree. I have succeeded in getting this patient to pronounce a few words, and he is making satisfactory progress in learning to write; from his being a tranquil patient, I hope, ultimately, I shall teach him to read.'

"Of the classes, the following are the arrangements:—

"'Reading the Testament twice a week.

"'Miscellaneous reading, ditto.

"'Writing, &c., three or four times a week.

"'Spelling, with slate lessons, ditto.

"'Oral instruction daily.

"'Singing with music in the chapel every Friday evening, the average number of male and female patients in attendance exceeding sixty.

"'Attendants' class for general improvement twice a week.

"'And lectures have been given on the following subjects to both male and female patients during the year:—On History and Geography—On the Geography of Plants—On the Pleasures arising from the Cultivation of Flowers—On the Shrubs and other Plants cultivated about the Grounds of the Asylum, with Descriptions of their properties and uses—On the Transformation and changes of Insects, illustrated by some beautiful diagrams, drawn and coloured by one of the patients. And on some occasions, above 150 were present at the lectures; and the attention with which they have listened to me was a sure indication how much they felt interested and amused, and I cannot but hope that in some instances they, indeed, were instructed.'

"The ingenuous and unpretending statement of Miss Waite will also indicate the difficulties of the undertaking, as well as its nature, and the very appropriate motives, and feeling sentiments, which amidst her very peculiar functions have been entertained.

"'Since the commencement of the school on the 1st of January, 1848, 73 female patients in the whole have attended.

"'Of these patients, Dr. Hitchman states that 33 are suffering from chronic mania, and liable to recurrent fits of violence; 32 are in various stages of imbecility; four are congenital idiots; and four belonged to the class of recent cases, and have left the asylum cured. Several in each of the first two classes are epileptic. Of these 73 patients—

| | | | |
|---|---|---|---|
| 10 | are above | 20 | and under 25 years of age. |
| 6 | under | 30 | |
| 11 | under | 35 | |
| 10 | under | 40 | |
| 19 | under | 45 | |
| 5 | under | 50 | |
| 7 | under | 55 | |
| 4 | under | 60 | |
| 1 | under | 65 | |

"'The patients are daily instructed in reading, writing, a variety of lessons on the black board, on natural history, spelling, &c.

"'The patients have continued to evince a desire to attend during the school hours, and according to their various capacities have benefited to a great extent. Many, whose time might have been passed in idleness, have been roused into watching, with something like interest, the patients who are disposed to exert themselves to a greater degree. Amongst the most interesting cases is one of a woman, aged 43, suffering from chronic mania, liable to periods of violence and epileptic. She first joined the school-classes in March, at that time scarcely able to read, and having no knowledge of writing: she now writes a good legible hand, and reads perfectly well; she has also improved in various ways. When in the school, she is tractable, obliging, and amiable, and so grateful for the new powers she has acquired, that it has been one of the happiest occupations to teach her.

"'M. B., 45, Chronic Mania. This patient always appears in a state of perfect unconsciousness, unless she is actually occupied; she has improved in reading, and has begun to learn writing.

"'A. D., 40, Chronic Mania. Has attended the school during the last six months, shows a great desire to improve, and has learned to read and write.

"'M. M., 42. A very violent patient, who, when she first attended the school, was rude and quarrelsome, but has become gentle and grateful: she has received a fair education, and is now most anxious to improve herself to the greatest extent.

"'M. B., 61, Chronic Mania. Has wished to attend the school the last two months; during that time, has been very punctual and remarkably persevering: she is improving greatly in reading and writing.

"'C. D., 40, Melancholia. A very singular patient, not often present in the school-room, and then almost constantly weeping: she has never spoken, but latterly has appeared willing to write. I trust she may be won to still greater exertions, which, it is hoped, will have the effect of causing her to forget for a time some real or imaginary sorrow.

"'Several patients, who had a very imperfect knowledge of reading, have sufficiently improved to read with ease. Many of the patients, not being able to read, have, since their attendance at the school, learned the alphabet, and are able to spell a few words. Other patients, of extremely feeble intellect, but not absolutely idiotic, have, with great difficulty, acquired a limited knowledge of the alphabet.

"'P. L., 43, Congenital Idiot. This is the most interesting case of that class. The patient, possessing a certain kind of energy and great love of approbation, has, encouraged apparently only by the latter feeling, acquired, with great labour, the knowledge of a few letters and a few simple words: she perfectly remembers, and is fond of repeating, many little facts connected with natural history, but has no idea of colour, form, or number. With the remaining three idiots, two of whom cannot articulate, all attempts at teaching up to this time have failed; but they have become obedient and cheerful, and anxious to attend the school.

"'There is an evening writing-class for those patients who are variously occupied during the day; 30 have been admitted, and on an average, 25 attend with great willingness, notwithstanding they have been actively engaged during many hours of the day.

"'Of the four patients who have been discharged cured, two, one aged 21, the other 27, made great progress, and expressed themselves very grateful for the opportunity given them of improvement.

"'The period since the school commenced has been too recent to enable me to offer to your notice many remarkable cases of improvement; but I have seen these heavily-afflicted women assemble in the school-room with cheerfulness and ready obedience, proceed to arrange themselves in their accustomed places, and apply themselves, with wonderful perseverance and diligence, to their several employments; and during the whole time they are present, the utmost order and decorum prevail, whilst the silence, unless they are called on to speak, is profound. And during the twelve months the school has been established, I can only remember one instance where it was necessary to request the attendant to remove a patient for violent conduct.

"'On the whole, my teaching has been received by the patients with such apparent pleasure and gratitude, that it has truly lessened whatever labour may attend it, and given me, with your sanction, every encouragement to go on rejoicing.' "

Nothing can be more gratifying than these details, particularly as the mental improvement would indicate, in some degree, the restoration of the intellectual faculties. We must now take farewell of the present Report. Whatever improvements the progress of science may suggest in the management of Hanwell Asylum, the committee of visitors is entitled to the thanks of the community for the zeal, assiduity, and humanity with which they have discharged their duties. And we cordially join them in their benevolent wish, that "long as the poor and helpless, suffering the worst of Heaven's visitation, need such asylums, may Hanwell remain for their reception and recovery. And may it, under the care of their successors, long continue to flourish and improve."

